# An Evaluation of the Bacterial Adherence to Casting Materials

**DOI:** 10.7759/cureus.16724

**Published:** 2021-07-29

**Authors:** Joseph E Massaglia, Cory Lebowitz, Keith Fitzgerald, Noreen J Hickok, Pedro Beredjiklian, Michael Rivlin

**Affiliations:** 1 Department of Orthopaedic Surgery, Rowan University School of Osteopathic Medicine, Stratford, USA; 2 Department of Orthopaedic Surgery, Thomas Jefferson University, Philadelphia, USA; 3 Division of Hand Surgery, Rothman Orthopaedic Institute, Philadelphia, USA

**Keywords:** microbiology, bacteria, casting, ‎3d printing, plastic

## Abstract

Introduction: The purpose of this study was to evaluate bacterial adherence to common casting materials including plaster of Paris (plaster), fiberglass, three-dimensional (3D) printed plastic, and silicone-coated 3D printed plastic.

Methods: The minimal inhibitory concentration of a phosphate-free detergent (Palmolive) needed to achieve total bacterial kill off was determined. 3D printed polylactic acid plastic samples were coated with silicone. Plaster, fiberglass, plastic, and silicone-coated plastic samples were inoculated with *Staphylococcus aureus*. After bacterial inoculation, scanning electron microscopy of the samples was performed to visualize bacterial adherence to the materials' surface. Using either sterile water or a 5% detergent solution, the materials were subjected to washings. Each material was run in 30 replicates: 6 without washing, 6 with sterile water for 1 minute, 6 with detergent for 1 minute, 6 with sterile water for 3 minutes, and 6 with detergent for 3 minutes. The replicates that did not undergo a washing trial represented the initial bacterial inoculation. Samples were then rinsed and sonicated in polysorbate to isolate the remaining adherent bacteria on the materials’ surface. The sonicated solutions were plated, incubated, and counted for quantification of colony forming units (CFU) of bacteria. This protocol was repeated for a total of four trials.

Results: During inoculation, there were significantly less bacteria that adhered to silicone-coated 3D printed plastic (58879 CFU) compared to plastic (217479 CFU), plaster (140063 CFU), and fiberglass (550546 CFU). Silicone coating showed further superiority. Silicone-coated 3D printed plastic was able to be decontaminated as demonstrated by significantly fewer remaining bacteria (9.3%) on its surface after being washed with a 5% detergent solution (1797 CFU) compared to sterile water (19321 CFU). The mean remaining bacteria on silicone-coated 3D printed plastic was significantly less than that remaining on all other materials when washed with either sterile water or a detergent solution for both durations of 1 minute and 3 minutes.

Conclusions: The current study demonstrates that significantly less bacteria adhere to the surface of 3D printed plastic with silicone coating showing added protection and that this material can be decontaminated to a greater degree with washing than conventional casting materials. These results provide evidence that 3D printed casts can be washed and successfully decontaminated during a patient’s period of immobilization, which is advantageous especially during an infectious crisis such as the coronavirus disease 2019 (COVID-19) pandemic.

## Introduction

Splinting and casting are often considered conservative treatment options for patients with extremity injuries. However, immobilization techniques that utilize fiberglass and plaster of Paris (plaster) material do not come without complications, including skin irritation, breakdown, and infection [1]. There have been reports of limb and life-threatening infections like toxic shock syndrome and necrotizing fasciitis in the pediatric population secondary to splint and cast immobilization [2]. Traditional splints and casts put patients at risk for cutaneous disease and are also a source of discomfort. The harbored flora contributes to odors associated with cast wear. For these reasons, there is an increasing clinical demand for a more hygienic, comfortable, and lightweight casting alternative that would effectively decrease the risk of these known complications.

Three-dimensional (3D) printing is becoming increasingly popular within the field of orthopaedics. This technology allows for the fabrication of custom-made, patient-specific casts that are effective in healing fractures with the added benefit of increased patient comfort and satisfaction [3-6]. Polylactic acid (PLA) plastic is a lightweight and waterproof thermoplastic polyester material commonly used to fabricate 3D printed casts. Acrylonitrile butadiene styrene (ABS) is another thermoplastic polymer material that is commonly used. Though a relatively new concept, evidence exists to support the increased use of 3D printed casts in everyday clinical practice for the nonoperative treatment of upper extremity injuries [3,4].

Little objective data exists to guide the choice of splinting and casting material. This selection may impact the risk of wound infection. At present, there is no evidence of one material being superior in bacterial colonization or contamination. Furthermore, no literature exists that compares the bacterial adherence to these different casting materials. The aim of this in vitro study is to evaluate the bacterial adherence to different casting materials including fiberglass, plaster, 3D printed plastic, and silicone-coated 3D printed plastic. We aim to determine the initial degree of bacterial contamination and the amount of bacteria remaining on these materials after washing trials with either a sterile water control or a detergent solution. We hypothesize that significantly less bacteria will adhere to silicone-coated 3D printed plastic during inoculation. We hypothesize that silicone-coated 3D printed plastic can be decontaminated as demonstrated by significantly less bacteria remaining on its surface after washing with a detergent solution compared to sterile water control. Furthermore, we hypothesize that significantly less bacteria will remain on silicone-coated 3D printed plastic compared to the other materials after all undergo washings with a detergent solution. 

This article was previously presented as a poster at the 2020 American Association for Hand Surgery (AAHS) Annual Meeting from January 8-11, 2020 and as a podium presentation at the 2020 American Society for Surgery of the Hand (ASSH) Annual Meeting from October 1-3, 2020.

## Materials and methods

Preparation of sample materials

**Figure 1:**
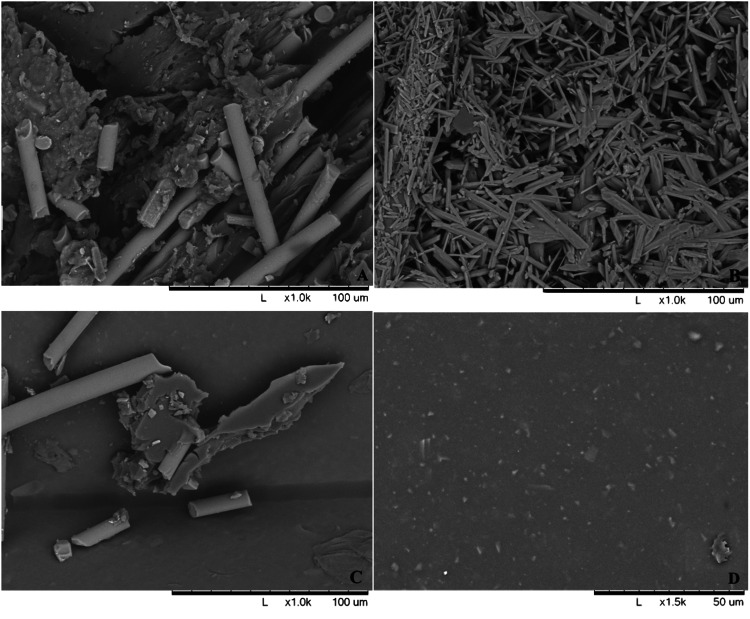
Scanning electron microscopy (SEM) of sample materials. This figure shows the SEMs displaying the surface topography of fiberglass (A), plaster (B), 3D printed plastic (C), and silicone coated 3D printed plastic (D) samples.

The four materials studied were fiberglass, plaster of Paris, 3D printed plastic, and silicone coated 3D printed plastic. Square-shaped samples (1.0 cm x 1.0 cm x 0.25 cm) of fiberglass and plaster were created by two authors (JM and KF). PLA plastic samples were printed to this same size using a commercially available, fused deposition modeling 3D printer (Ultimaker 3 Ex, Geldermalsen, Netherlands) with a layer resolution of 20-200 microns and an XYZ accuracy (dimensional accuracy of the 3D printer in the x-axis, y-axis, and z-axis, respectively) of 12.5/12.5/2.5 microns. A portion of these plastic samples was dipped into a liquid silicone mix and hung, open to room air, allowing them to harden. Excess silicone was then sharply removed so that the entire square-shaped sample was evenly coated with a thin layer of silicone to ensure each sample was nearly the same size. Scanning electron microscopy (SEM) of a sample of each material was performed in order to evaluate its surface topography (Figure 1).

Description of breakpoint trial

A breakpoint trial was performed to determine the minimal inhibitory concentration (MIC) of a detergent solution that would achieve bacterial kill off as defined by lack of turbidity in an overnight culture of *Staphylococcus aureus* strain ATCC 25923 (ATCC, Manassas, VA, USA) and also by the ability of the detergent solution to reduce bacterial numbers to approximately 0 colony forming units (CFU)/mL. Specifically, using a single colony, ATCC 25923 was added to 5 mL of Trypticase Soy Broth (TSB) and incubated overnight. To restore rapid growth, the overnight culture was diluted 1:2 in TSB and cultured for 1 hour at 37°C and 190 revolutions per minute (RPM). The subculture was diluted to ~106 CFU/mL using turbidity as compared to a 0.5 McFarland standard.

Detergent solutions (0%, 0.1%, 1%, 5%, 10%, 20% weight:volume) were created using a phosphate-free detergent (Palmolive) and Mueller Hinton Broth (MHB). This detergent was chosen as it is readily available to patients for use in their own homes. The detergent solution (180 µL) was combined with the standardized bacterial solution (20 µL) in a 96-well V-bottom plate and incubated overnight at 37°C. Bacteria were pelleted at 2272 RPM for 5 minutes using a rotor for plates, washed with 200 µL of phosphate-buffered saline (PBS), pelleted again, and resuspended in 200 µL of PBS. Suspensions were serially diluted, plated on 3M Petrifilm (3M, St. Paul, MN, USA) grids, and after overnight incubation at 37°C, counted where a countable range was defined as 20-500 CFU. Each concentration of detergent solution had 6 replicates and the entire protocol was repeated three separate times. The MIC of the detergent solution was observed to be 0.1%.

Description of a washing trial

The washing conditions tested included 0 minute with sterile water (bacterial inoculation), 1 minute with sterile water, 1 minute with 5% detergent solution, 3 minutes with sterile water, and 3 minutes with 5% detergent solution. Six individual samples of each material were subjected to a single washing condition; therefore, 30 samples of each material were used in a single trial. Samples were placed into 24-well plates.

**Figure 2:**
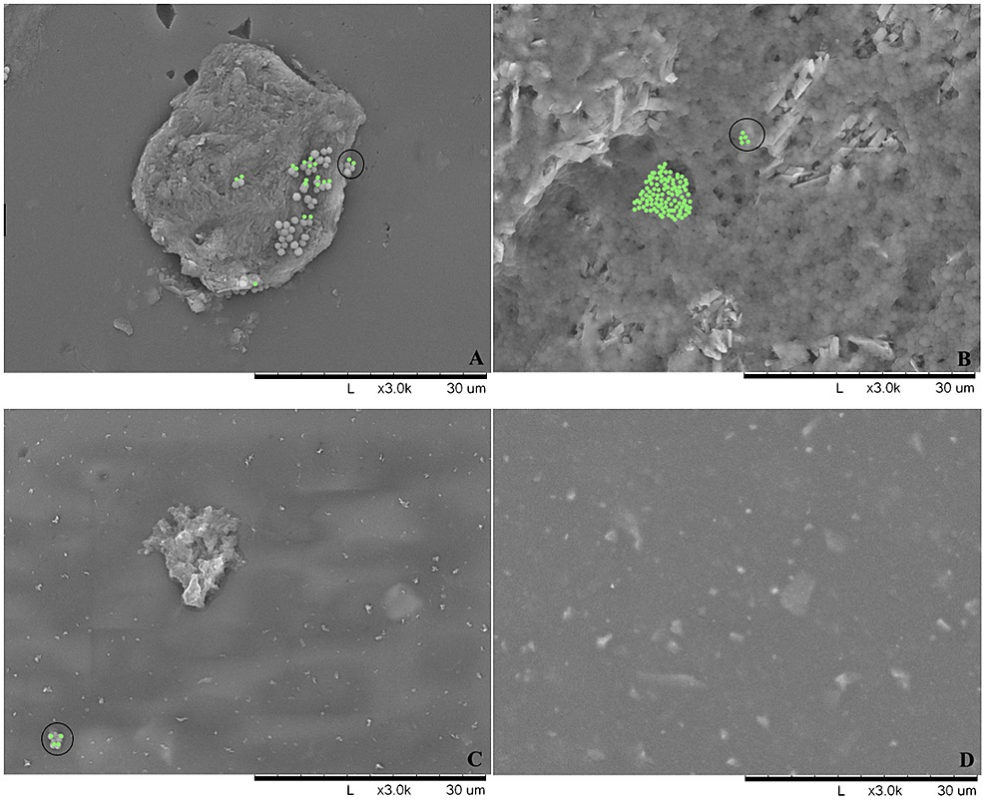
Scanning electron microscopy (SEM) of sample materials after bacterial inoculation. This figure shows the SEMs of the samples after bacterial inoculation with *Staphylococcus aureus*, ATCC 25923 (colony = green highlight; bacterial density = circle) on the surface of fiberglass (A), plaster (B), 3D printed plastic (C), and silicone coated 3D printed plastic (D).

A standardized bacterial solution of ATCC 25923 at a concentration of 107 CFU/mL was created. The bacterial number was determined based on comparison to a 0.5 McFarland standard. The concentration of 107 CFU/mL was chosen based on previous experience incubating 3D material in tissue culture multi-well plates. Two mL of the standardized ATCC 25923 solution was added to each well in order to submerge the sample and incubated for 1 hour in a standing 37°C incubator. After bacterial inoculation, one sample of each material underwent SEM (Figure 2). The 6 samples of each material that did not undergo a washing trial (0 minute with sterile water) were transferred to a clean 24-well plate. All other samples were then transferred to bottles containing either the sterile water control or the 5% detergent solution. The bottles were placed on a shaker set to 80 RPM for either 1 minute or 3 minutes depending on the washing condition being tested.

Samples were then washed twice with sterile water and transferred to clean 24-well plates containing 1 mL of 0.3% Tween-20 (Fisher Scientific, Waltham, MA, USA). Adherent bacteria were removed from the samples’ surface and suspended in solution via sonication in the non-ionic Tween-20. The combination of detergent in PBS and sonication allows for a greater retrieval of the adherent bacteria from the samples’ surface. Sonication times vary between different devices. We chose ours based on testing planktonic bacteria in the presence of the 0.3% Tween-20 to determine when bacterial numbers started to decrease, indicating lysis. All plates were sonicated for 15 minutes to suspend the adherent bacteria. The time chosen is slightly less than that when deleterious effects of the sonication start to occur. The suspended solutions of bacteria were diluted with PBS, plated on 3M Petrifilm grids, incubated overnight at 37°C, and counted. The outcome was defined as the remaining CFU on the samples’ surface as determined by the enumeration of bacterial counts on the 3M Petrifilm. The bacterial counts were performed by the same author (KF) who ran the experimental trials.

This entire protocol was repeated 3 additional times for a total of 4 washing trials. In total, 120 samples of each material were tested: 24 without treatment after bacterial inoculation (0 minute with sterile water), 24 with sterile water for 1 minute, 24 with 5% detergent solution for 1 minute, 24 with sterile water for 3 minutes, and 24 with 5% detergent solution for 3 minutes.

Statistical analysis

Continuous variables were reported as means and standard deviations and compared using either a two-tailed independent t-test or single-factor ANOVA as the data was normally distributed. A p-value of less than 0.05 was considered statistically significant. Post hoc analysis testing was performed to confirm the statistical significance of our comparisons. A Dixon’s Q test identified and rejected outliers and after the removal of these data points, a power analysis was performed yielding a beta value of 0.99. Our study was powered with the pre-determined sample size.

## Results

The SEM images of each materials’ surface before and after bacterial inoculation are shown in Figure 1 and Figure 2, respectively. The surface topography of each material differs. Notice how the surface of plaster (B) and to a lesser degree fiberglass (A) are covered in a sea of biofilm with individual bacterial colonies, highlighted green, nestled within the crevices and imperfections of the porous materials. The amount of bacteria that adhered to each materials’ surface during bacterial inoculation is represented by the 0 minute sterile water washing condition (Table 1). During inoculation, there were significantly less bacteria that adhered to the silicone-coated 3D printed plastic compared to all other materials (p=0.002). When compared directly to fiberglass, there were significantly less bacteria that adhered to 3D printed plastic (p=0.037) and silicone coated 3D plastic (p=0.002) during inoculation.

**Table 1:** The amount of bacteria adhering to each material’s surface during bacterial inoculation CFU=colony forming units, SD=standard deviation, min=minute. *Silicone coated 3D printed plastic, **3D printed plastic

Washing	Material	Average CFU ± SD	P-value
0 min sterile water	Silicone*	5.8879 x 10^4^ ± 6.7112 x 10^4^	0.002
	Plastic**	2.17479 x 10^5^ ± 4.94838 x 10^5^	
	Fiberglass	5.50546 x 10^5^ ± 7.41340 x 10^5^	
	Plaster	1.40063 x 10^5^ ± 1.20262 x 10^5^	

The ability to decontaminate silicone-coated 3D printed plastic was evaluated by comparing the number of remaining bacteria on the material’s surface after being washed with 5% detergent versus a sterile water control. This was evaluated for both 1 minute and 3 minute washing conditions (Table 2). Silicone-coated 3D printed plastic was able to be decontaminated as demonstrated by significantly fewer remaining bacteria on its surface after being washed with 5% detergent compared to a sterile water control for both 1 minute (p=0.020) and 3 minutes (p=0.014).

**Table 2:** The amount of bacteria remaining on the surface of silicone-coated 3D printed plastic after washing conditions CFU=colony forming units, SD=standard deviation, min=minute. *Silicone-coated 3D printed plastic

Material	Washing	Average CFU ± SD	P-value
Silicone*	1 min sterile water	1.9321 x 10^4^ ± 3.4379 x 10^4^	
	1 min 5% detergent	1.797 x 10^3^ ± 1.205 x 10^3^	0.020
	3 mins sterile water	3.3088 x 10^4^ ± 5.6931 x 10^4^	
	3 mins 5% detergent	1.338 x 10^3^ ± 3.138 x 10^3^	0.014

**Figure 3:**
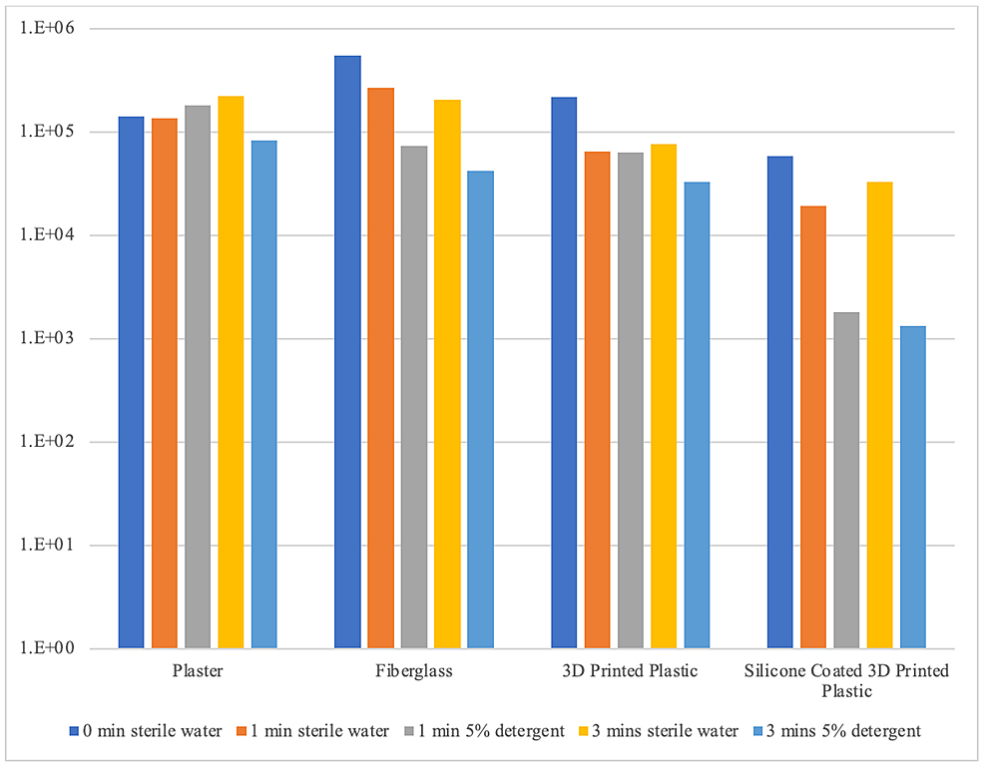
The quantity of bacteria on materials' surface after washing trials This graph shows the mean remaining colony forming units CFU (y-axis) on the casting materials’ surface (x-axis) after bacterial inoculation (0 min sterile water) and washing trials with sterile water and a 5% detergent solution for 1 minute and 3 minutes.

The amount of bacteria that adhered to each materials’ surface during inoculation and the amount remaining after all washing conditions are displayed in Figure 3.

The remaining bacteria on silicone-coated 3D printed plastic’s surface was significantly less than that remaining on all other materials’ surface when washed with either sterile water for 1 minute (p=0.009) or 3 minutes (p=0.014) or 5% detergent for either 1 minute (p<0.001) or 3 minutes (p<0.001) (Table 3). When compared directly to fiberglass, silicone-coated plastic is decontaminated to a greater degree when washed with 5% detergent for either 1 minute (p=0.001) or 3 minutes (p<0.001). However, there is no difference in the degree of decontamination between fiberglass and plastic when washed with 5% detergent for either 1 minute (p=0.367) or 3 minutes (p=0.253). 

**Table 3:** The amount of bacteria remaining on each material’s surface after all washing conditions CFU=colony forming units, SD=standard deviation, min=minute. *Silicone coated 3D printed plastic, **3D printed plastic

Washing	Material	Average CFU ± SD	P-value
1 min sterile water	Silicone*	1.9321 x 10^4^ ± 3.4379 x 10^4^	0.009
	Plastic**	6.5088 x 10^4^ ± 9.7281 x 10^4^	
	Fiberglass	2.69183 x 10^5^ ± 5.02924 x 10^5^	
	Plaster	1.35292 x 10^5^ ± 1.21433 x 10^5^	
1 min 5% detergent	Silicone*	1.797 x 10^3^ ± 1.205 x 10^3^	< 0.001
	Plastic**	6.3141 x 10^4^ ± 9.8257 x 10^4^	
	Fiberglass	7.2965 x 10^4^ ± 1.00660 x 10^5^	
	Plaster	1.79188 x 10^5^ ± 2.22669 x 10^5^	
3 mins sterile water	Silicone*	3.3088 x 10^4^ ± 5.6931 x 10^4^	0.014
	Plastic**	7.5985 x 10^4^ ± 2.20065 x 10^4^	
	Fiberglass	2.04972 x 10^5^ ± 2.57903 x 10^5^	
	Plaster	2.22250 x 10^5^ ± 3.16534 x 10^5^	
3 mins 5% detergent	Silicone*	1.338 x 10^3^ ± 3.138 x 10^3^	< 0.001
	Plastic**	3.2705 x 10^4^ ± 3.8241 x 10^4^	
	Fiberglass	4.1705 x 10^4^ ± 5.3276 x 10^4^	
	Plaster	8.1992 x 10^4^ ± 8.9638 x 10^4^	

## Discussion

In light of the current pandemic, new protocols are advising for less physician-patient contact, shortening the contact time, and maintaining a safe distance in order to decrease the risk of spreading the novel coronavirus disease 2019 (COVID-19). In an effort to decrease follow-up for cast removal, recommendations exist to avoid unnecessary fiberglass casting when alternative methods can be utilized. Custom-made 3D printed casts can be ordered remotely, delivered directly to patients’ homes, and applied and removed by the patient without ever having to physically enter a physician’s office. These casts can be washed and successfully decontaminated during a patient’s period of immobilization, which is advantageous during an infectious crisis such as the COVID-19 pandemic.

It is postulated that the success of detergent solutions at removing bacteria from material surfaces can be attributed to its physical surfactant-like properties rather than its antimicrobial ones. The current study supports these findings by demonstrating that significantly fewer bacteria remain on the surface of silicone-coated 3D printed plastic after being irrigated with a detergent solution compared with sterile water. The detergent solution we chose is a household product commercially available to patients.

Within the field of orthopaedics, a variety of biomaterials are placed on or implanted into the body. In vitro models have shown that the biomaterial’s surface topography, its roughness, is a critical variable for bacterial adherence. Yoda et al. evaluated the ability of *Staphylococcus epidermidis* to adhere to the surface of solid materials that possessed differing levels of roughness [7]. Disc-shaped samples of five metals were polished with or without subsequent abrasion. Scanning electron microscopy (SEM) of the samples’ surface was performed in order to confirm a statistically significant difference between the average surface roughness of the fine and coarse group. These samples were then sterilized and inoculated with a standardized bacterial solution of *Staphylococcus epidermidis*. Samples were rinsed to remove unbound and deposited cells and then placed in a fresh broth medium for sonication in order to remove all adherent bacteria. The resulting sonicated suspensions were cultured and incubated prior to the quantification of bacterial CFU. This experimental design was very similar to the one utilized in the current study. The authors were able to demonstrate that statistically significant greater amounts of *Staphylococcus epidermidis* adhered to each sample in the coarse group compared to the fine group. They concluded that surface roughness not only increases surface area but also provides a scaffold for bacterial adhesion. Like plastic and silicone, fiberglass is a waterproof material, however, our results demonstrate that significantly more bacteria adhere to it and it is not decontaminated to the same degree as silicone. This is likely due to the porous nature of fiberglass providing a larger surface area for bacterial adhesion. Bacteria colonize between the crevices and imperfections of fiberglass and plaster, making these materials more resistant to decontamination compared to the smooth surface of plastic and silicone (Figure 2). The current study is in accordance with those prior supporting a positive correlation between early bacterial adherence and surface roughness [7].

The current study had a number of limitations. First, the fiberglass and plaster samples’ size varied slightly compared to the precise, 3D printed 1.0 cm x 1.0 cm x 0.25 cm plastic samples. All attempts were made to make each fiberglass and plaster sample the exact same size as the plastic samples. Because these samples were measured and cut by two independent investigators, there is a human inaccuracy that contributed to the small differences in the size of the material samples. These size differences matter because the quantity of bacteria that adhere to an individual sample is dependent on its surface area, amongst other things, which varies based on its size. However, in order to make the differences in sample size near negligible, a heavily concentrated bacterial solution was utilized during inoculation. The absolute difference in the quantity of CFU adhering to a sample’s surface secondary to its size is not on the same magnitude as the bacterial solution’s concentration of 10^7^ CFU/mL. The size variation is likely in both directions and a large number of samples were studied, further minimizing the effect of this factor. Second, the enumeration of bacterial counts on 3M Petrifilm after overnight incubation was performed by a single investigator once and thus there is no intra- or inter-observer reliability to report. But, the absolute difference in CFU remaining on silicone coated plastic is so large in all washing trials that a second investigator’s observance would not likely overcome the statistical significance. Third, despite our washing protocol being very exact and reproducible in an in vitro experimental setting, it does not translate to a practical scenario where patients would be using an inconsistent amount of detergent to scrub their casts for an unpredictable length of time. Despite these limitations, it was possible to make a simple comparison of bacterial adhesion on various material surfaces used in clinical practice. Additionally, waterproof fiberglass padding adds a layer where bacteria can get trapped. With 3D printed casts a padding layer is not essential. Thus, no additional layer likely leads to superior decontamination of these materials further strengthening this study’s conclusions. As the use of 3D printed casts becomes more common, future studies should focus on defining the decreased relative risk of underlying skin complications compared to fiberglass and plaster casts.

## Conclusions

Prior to this study, the degree of bacterial colonization of traditional casting materials in addition to 3D printed plastic had not been evaluated. This study demonstrates that significantly fewer bacteria adhere to the surface of 3D printed plastic with added silicone layer showing further improvement in this property. Furthermore, silicone-coated plastic can be decontaminated to a greater degree when washed with a detergent solution. The current study supports the clinical utility and potential benefits of 3D printed plastic casts by proving the material’s superiority in bacterial colonization and contamination at the fundamental microbiologic level.
